# Increased Mercury Levels in Patients with Celiac Disease following a Gluten-Free Regimen

**DOI:** 10.1155/2015/953042

**Published:** 2015-02-23

**Authors:** Luca Elli, Valentina Rossi, Dario Conte, Anna Ronchi, Carolina Tomba, Manuela Passoni, Maria Teresa Bardella, Leda Roncoroni, Gianpaolo Guzzi

**Affiliations:** ^1^Center for the Prevention and Diagnosis of Celiac Disease, Gastroenterology and Endoscopy Unit, Fondazione IRCCS Ca' Granda Ospedale Maggiore Policlinico, Department of Pathophysiology and Transplantation, Università degli Studi di Milano, Via Francesco Sforza 35, 20122 Milan, Italy; ^2^Italian Association for Metals and Biocompatibility Research (AIRMEB), Via Banfi 4, 20122 Milan, Italy; ^3^Pavia Poison Control Center and National Toxicology Information Centre, Toxicology Unit, IRCCS Maugeri Foundation and University of Pavia, Via Salvatore Maugeri 10, 27100 Pavia, Italy; ^4^Department of Biomedical, Surgical and Dental Sciences, Università degli Studi di Milano, Via Festa del Perdono 7, 20122 Milan, Italy

## Abstract

*Background and Aim*. Although mercury is involved in several immunological diseases, nothing is known about its implication in celiac disease. Our aim was to evaluate blood and urinary levels of mercury in celiac patients. *Methods*. We prospectively enrolled 30 celiac patients (20 treated with normal duodenal mucosa and 10 untreated with duodenal atrophy) and 20 healthy controls from the same geographic area. Blood and urinary mercury concentrations were measured by means of flow injection inductively coupled plasma mass spectrometry. Enrolled patients underwent dental chart for amalgam fillings and completed a food-frequency questionnaire to evaluate diet and fish intake. *Results*. Mercury blood/urinary levels were 2.4 ± 2.3/1.0 ± 1.4, 10.2 ± 6.7/2.2 ± 3.0 and 3.7 ± 2.7/1.3 ± 1.2 in untreated CD, treated CD, and healthy controls, respectively. Resulting mercury levels were significantly higher in celiac patients following a gluten-free diet. No differences were found regarding fish intake and number of amalgam fillings. No demographic or clinical data were significantly associated with mercury levels in biologic samples. *Conclusion*. Data demonstrate a fourfold increase of mercury blood levels in celiac patients following a gluten-free diet. Further studies are needed to clarify its role in celiac mechanism.

## 1. Introduction

Mercury (Hg) is ubiquitous environmental heavy metal, naturally originating from erosion of the volcanic rocks and accumulating in the food chain. Besides its natural presence, Hg environmental concentration is progressively increasing due to its employ in human industry and manufactures (medications, thermometers, blood-pressure cuffs, batteries, switches, and fluorescent light bulbs) [1–4]. Thus, Hg is actually considered a pollutant and, due to its deleterious effects on humans, it is generally considered toxic especially for the nervous system [5]. Mercury main sources for human being are represented by fish consumption, dental amalgams, and vaccines [4, 6]. Once in the body, Hg atoms bind the proteic thiol groups and deposit in all tissues, where they can remain for a long time, triggering its chronic consequences on health [7, 8]; in fact, behind the Hg acute intoxication syndrome (exemplified by Minamata Bay disaster) [9], recent scientific advances have demonstrated that Hg is a cofactor in several multifactorial diseases (cardiovascular, neurodegenerative, and autoimmune), as a consequence of its biological effect on inflammation and immune system [1, 10–14]. Hg is mainly an HLA class II-restricted immunostimulator, leading to the proliferation of B and T lymphocytes and formation of autoantibodies and immunocomplexes [15]. In the last decades, the concomitant increase of autoimmune disorders and Hg environmental pollution represents an intriguing point [16]. In particular, the hypothesis of Hg-autoimmunity connection appears plausible for disorders characterized by a HLA restricted genetic background [17, 18] as celiac disease (CD), an HLA class II-dependent autoimmune disease of the small bowel [19–21]. CD is a common (prevalence rate 1 : 100) chronic enteropathy triggered, in genetically predisposed subjects (carrying the HLA DQ2 and/or DQ8 haplotypes), by the ingestion of gluten [19, 22, 23]. In CD, gluten induces and fuels an immunological response inducing a small bowel mucosa damage characterized by intraepithelial lymphocytosis, crypt hyperplasia, and villous atrophy [24]. However, other-than-gluten environmental factors are supposed to be present in the development of CD. Among them, infectious agents (virus) [25] and microbiota [26] have been evaluated without conclusive data.

In this context, considering the absence of pertinent findings, Hg role in the CD pathomechanism could be hypothesized.

The present study aimed to evaluate the Hg levels in CD patients.

## 2. Materials and Methods

### 2.1. Patients

From January 1, 2007, to June 6, 2010, subjects signing an informed consent were consecutively and prospectively enrolled at the “Center for Prevention and Diagnosis of Celiac Disease” of the “Fondazione IRCCS Ca' Granda Ospedale Maggiore Policlinico,” Milan, Italy. CD diagnosis was based on the presence of serological anti-tissue transglutaminase (tTGA, ELISA, or radioimmunoassay tests) and/or anti-endomisium (EmA, immunofluorescence technique) IgA antibodies and a duodenal histology presenting villous atrophy (grade 3 according to the Marsh-Oberhuber classification) [27]. The enrolment included both newly diagnosed and treated (following a gluten-free diet, GFD) CD patients. Treated CD patients were compliant to the GFD with normalization of serological tests and restoration of the duodenal villous architecture (grade 0 according to the Marsh-Oberhuber classification). A group of non-CD subjects was enrolled as controls. Patients reporting an occupational exposure to Hg were excluded such as patients reporting renal or liver pathologies. To avoid environmental pollution differences, all participants were resident in the same urban area (Milan, Northern Italy).

Enrolled patients underwent an odontostomatologic visit to evaluate dental amalgam fillings and completed an operator assisted questionnaire investigating the intake of potential Hg containing food (fish, days of intake/month), the presence of possible factors influencing the Hg levels (nocturnal bruxism, chewing gum use in presence of Hg amalgams), and the presence of symptoms possibly correlated with Hg exposure (metallic taste, foggy mind, chronic fatigue, and tremor) [28]. Moreover, a detailed seven-day alimentary diary was completed by participants.

The study was approved by the ethical committee of the “Fondazione IRCCS Ca' Granda Ospedale Maggiore Policlinico.”

### 2.2. Mercury Analysis of Biological Samples

Total Hg levels were assessed by using flow injection inductively coupled plasma mass spectrometry (FI-ICP-MS) as previously described [29, 30]. Briefly, at enrollment, fasting morning venous peripheral whole blood samples (4 mL) were collected in Hg-free polypropylene tubes containing potassium EDTA, as an anticoagulant. First morning urine specimens (100 milliliters) were obtained and stored at +4°C until mercury analysis. Both blood and urine samples were delivered immediately to the laboratory of toxicology for FI-ICP-MS Hg analysis and were processed within 24–72 hours after collection. The limit of detection was 0.05 micrograms per liter. Internal and external quality-control procedures were done.

### 2.3. Statistical Analysis

All the assumptions were verified using SPSS version 18 (IBM SPSS, Italy), and a *P* value < 0.05 was considered statistically significant (significance level of the tests 5%, two tails). Continuous variables were analyzed with the ANOVA one-way variance test, Turkey's test, or the nonparametric Kruskal-Wallis test when indicated. Categorical variables were compared by *χ* ^2^ or Fisher's exact test. Kolmogorov-Smirnov was used to assess Gaussian distribution of the data. Correlations were analysed by Pearson or Spearman test in case of Gaussian or nonparametric variables.

## 3. Results

Thirty CD patients (20 treated and 10 untreated) and 20 healthy controls were enrolled. As shown in Table 1, patient age, sex, weight, and height (body mass index, BMI) were not statistically different among the three investigated groups.

**Figure 1:**
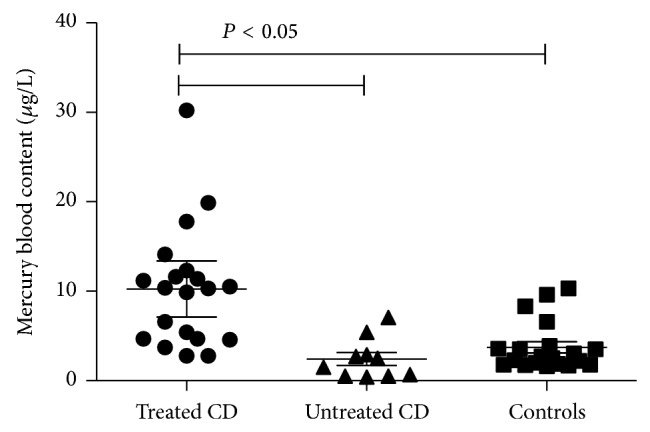
Mercury (Hg) blood levels of untreated and treated celiac (CD) patients and healthy controls. Mean, 95% confidence intervals, and statistical significance are reported in the plot.

Details of blood and urinary Hg levels of the analysed groups are reported in Table 2. Mercury blood levels resulting significantly increased in treated CD patients compared to untreated CD, as detailed in Figure 1.

The number of Hg amalgam fillings was 5.0 ± 2.9, 3.0 ± 2.8, and 3.8 ± 2.6 in untreated CD, treated CD, and healthy controls, respectively, without a statistical difference among the groups. The number of amalgam fillings was unrelated to both blood and urinary Hg levels (unreported data). Bruxism prevalence and chewing gum use were comparable in the three aforementioned groups (five, nine, and ten cases affected by bruxism and two, three, and four chewing gum users in untreated CD, treated CD, and healthy controls, resp.).

A part from for the presence of gluten-free products in the diet of treated CD, the weekly intake of fish and seafood, nonalcoholic beverages, and composite food (i.e., the main sources of dietary Hg), as obtained from the seven-day long questionnaire, was comparable in the studied groups and did not correlate with the Hg levels in both blood and urine. In particular the fish intake ranged from 3 to 4 times per week in the investigated groups (data not shown).

As showed in Figure 1, the group of treated CD patients could be divided into two subgroups, the first composed of 11 patients with Hg blood levels ≥10 *μ*g/L and the second composed of 9 patients with blood Hg levels <10 *μ*g/L. Among treated CD patients with Hg blood levels ≥10 *μ*g/L, metallic taste was reported by 36% of subjects versus 33% of patients with Hg blood levels <10 *μ*g/L, foggy mind 45% versus 11%, chronic fatigue 18% versus 0%, and tremor 27% versus 22%. Although the above Hg-related symptoms were increased in group with blood Hg levels ≥10 *μ*g/L, differences were not statistically significant probably due to the limited number of cases.

## 4. Discussion

The present study demonstrated an increase of Hg levels in patients affected by CD following a GFD. This finding, presented here for the first time, deserves some comments and considerations.

As for many other autoimmune diseases, CD prevalence is increasing in the last decade [16, 31]. In the past, CD was considered a rare disorder of childhood, characterised by malabsorption and growth deficiency [32]; nowadays, it is the most frequent autoimmune chronic enteropathy (1 : 100) in western countries and it can be diagnosed at every age [19, 33, 34]. This “pandemia” is partially explained by the improvement of diagnostic tests (ELISA commercial kits for detection of anti-tissue transglutaminase antibodies) and digestive endoscopy worldwide diffusion. A “real” CD increase seems demonstrated by studies based on biobanked biologic samples [35, 36]. This finding is difficult to explain but it is clear that unknown environmental factors are involved as suggested by studies on monozygotic twins demonstrating 80% concordance rate for the development of CD [37]. Celiac disease pathogenetic cascade is started by an activation of mucosal T cells, leading to a chronic autoimmune reaction responsible for the typical duodenal damage with T cell infiltration, crypt hyperplasia, and villous atrophy [38]. This mechanism occurs exclusively in the presence of the HLA DQ2 and/or DQ8 haplotypes [38]. During the last years, different researchers investigated factors potentially stimulating the onset of an overt CD or simply increasing its risk [25, 39–41]. Previous studies investigated breast feeding habit [42], virus infection (rotavirus) [25], and the intake of high gluten-containing grain or the use of enzymes (bacterial transglutaminases) in food industry [23], without conclusive data. No environmental pollutant or toxicants have been investigated in CD, although environmental pollution is getting higher and higher in the last decades. Different studies demonstrated that Hg participates in the development of several immune disorders as membraneous nephropathy [43], autoimmune glomerulonephritis [44], Wegener's granulomatosis [45], scleroderma [46], systemic lupus erythematosus [47], pemphigus [48], and multiple sclerosis [49]. Epidemiologic data suggest that Hg amalgam fillings have an effect in the exacerbation and/or onset of multiple sclerosis [47]. This connection is strengthened by the clinical improvements reported by patients affected by Hashimoto's thyroiditis after Hg amalgam fillings removal [47]. Again, Hg induces in humans the formation of different types of autoantibodies (antinuclear, antinucleolar, anti-fibrillarin, and anti-laminin) and its effect is particularly relevant in diseases controlled by the major histocompatibility complex (MHC) class II (as CD), although these data mainly come from animal studies [1, 13, 50].

Another important point is represented by the increase of Hg levels after duodenal mucosa normalization in treated CD. This finding represents the first demonstration that duodenal atrophy could lead to a reversible Hg “malabsorption.” Actually, very few are known about the Hg transport/absorption in human small bowel. Recent* in vitro* data on Caco-2 cell line suggest the existence of an active Hg transport into enterocytes although the transporter remains unknown [51]. This “scenario” could be comparable to that observed for iron intestinal transport when a genetically determined hemochromatosis is present in association with CD: the concomitant duodenal atrophy preserves patients by iron overload and the development of clinically relevant hemochromatosis, usually developing during the GFD (i.e., duodenal normalization) [52, 53]. Interpretation of the present finding leads to important observations: (i) intestinal Hg absorption is mainly localized in the duodenum; (ii) Hg seems to be absorbed by transporters mainly localised on the apical part of the villi, typically damaged in CD. However, another factor increasing the Hg levels could be the GFD itself; in fact, GFD could have a positive effect favoring the Hg release from the intracellular stores to extracellular fluids (i.e., blood). If this process could be related to the presence of symptoms (metallic taste, foggy mind, chronic fatigue, and tremor) resembling Hg poisoning as detected in our cohort, it remains an interesting point to be investigated in large series of patients.

The relevant increase of Hg blood levels in treated CD is not justified by differences in seafood consumption and dental amalgam fillings, which are considered important sources of Hg for humans by the World Health Organization, WHO [11]. Fish generally accumulates Hg through the alimentary chain and for these reasons fish-eating fishes (swordfish, tuna, etc.) are more likely Hg-contaminated, while amalgam fillings continuously release Hg in the mouth, especially in patients affected by bruxism or using chewing gum [11].

Looking at these data, Hg accumulation could also be due to a genetic predisposition of CD subjects to retain it (see also low Hg levels in urine). In our study we analysed whole blood and urine Hg levels, believed to be reliable marker for Hg exposure; however this choice could have some implications. When exposure continues, tissue levels of Hg in humans are increased, mainly in brain (pituitary gland and cerebral cortex), central nervous system, thyroid, and kidneys. Hence, concentrations of Hg in blood and urine may underestimate retention of Hg in the organism. In other words, there is the possibility that measurements of Hg in blood and urine do not fully reflect the real body content.

Another limitation of the study, besides the limited number of patients, is that blood and urine Hg concentrations not always correlate with the noxious effects in humans. In fact, response to Hg is influenced by different factors inducing the onset of the immune or neurologic alteration. These factors are largely unknown but genetic ones are the strongly suspected: subjects with a predisposition to accumulate Hg in determined cells or tissues could be more susceptible to specific toxic effects [4].

In conclusion, our study demonstrates an alteration of Hg content in CD when a gluten-free regimen is followed. This result could be due to an altered response to Hg exposure, with the tendency to accumulate it. Further studies are needed to clarify if CD genetic background could generate “sensitivity” to Hg proinflammatory effect and inspire new rules for the surveillance of Hg content in food.

## Figures and Tables

**Table 1:** Clinical and demographic parameters of the enrolled subjects.

	Untreated CD (*n* = 10)	Treated CD (*n* = 20)	Healthy controls (*n* = 20)	*P*
Age (years)	40.4 ± 7.5	40.1 ± 9.7	39.6 ± 10.9	NS
Male/female	3/7	3/17	4/16	NS
Weight (Kg)	57.0 ± 11.2	60.2 ± 8.4	62.1 ± 10.4	NS
Height (cm)	167.7 ± 5.0	165.7 ± 6.6	169.8 ± 9.0	NS
BMI	20.2 ± 3.6	21.9 ± 3.0	21.8 ± 2.8	NS
tTGA (positive %)	100	0	0	NA
Villous atrophy (Pts %)	100	0	0	NA
GFD (years)	NA	8.2 ± 8.2	NA	NA

BMI: body mass index; CD: celiac disease; NS: not significant; NA: not applicable; GFD: gluten-free diet; tTGA; tissue transglutaminase antibodies; Pts: patients.

**Table 2:** Urinary and blood mercury (Hg) levels in celiac patients and healthy controls.

	Untreated CD (*n* = 10)	Treated CD (*n* = 20)	Healthy controls (*n* = 20)
Hg blood (*μ*g/L)	2.4 ± 2.3	10.2 ± 6.7^*^	3.7 ± 2.7
Hg urine (*μ*g/L)	1.0 ± 1.4	2.2 ± 3.0	1.3 ± 1.2

CD: celiac disease; Hg: mercury.

^*^ *P* < 0.05 versus Hg blood levels of untreated CD and healthy controls.
